# Publisher Correction: Efficient plasmon-hot electron conversion in Ag–CsPbBr_3_ hybrid nanocrystals

**DOI:** 10.1038/s41467-019-09520-3

**Published:** 2019-03-27

**Authors:** Xinyu Huang, Hongbo Li, Chunfeng Zhang, Shijing Tan, Zhangzhang Chen, Lan Chen, Zhenda Lu, Xiaoyong Wang, Min Xiao

**Affiliations:** 1 National Laboratory of Solid State Microstructures, School of Physics, and Collaborative Innovation Center of Advanced Microstructures, Nanjing University, 210093 Nanjing, China; 2 College of Engineering and Applied Sciences, Nanjing University, 210093 Nanjing, China; 3 Synergetic Innovation Center in Quantum Information and Quantum Physics, University of Science and Technology of China, 230026 Hefei, Anhui China; 4 Hefei National Laboratory for Physical Sciences at the Microscale, and Department of Chemical Physics, University of Science and Technology of China, 230026 Hefei, Anhui China; 5 Department of Physics, University of Arkansas, 72701 Fayetteville, AR USA; 6 Present Address: Key Laboratory of Flexible Electronics (KLOFE) and Institute of Advanced Materials (IAM), Jiangsu National Synergetic Innovation Center for Advanced Materials (SICAM), Nanjing Tech University, 30 South Puzhu Road, 211816 Nanjing, China

Correction to: *Nature Communications* 10.1038/s41467-019-09112-1, published online 11 March 2019

The original version of this Article incorrectly acknowledged Zhangzhang Chen as a corresponding author instead of Chunfeng Zhang. This has now been corrected in both the PDF and HTML versions of the Article.

