# Supplementary material for: Efficient plasmon-hot electron conversion in Ag–CsPbBr3 hybrid nanocrystals
Source: Nat Commun. 2019 Mar 11;10:1163. doi: 10.1038/s41467-019-09112-1 (PMC6411736; doi:10.1038/s41467-019-09112-1)
Supplement: Supplementary file 1 — Supplementary Information [file 41467_2019_9112_MOESM1_ESM.pdf]

# Efficient plasmon-hot electron conversion in Ag–$CsPbBr_3$ hybrid nanocrystals

Huang et al.

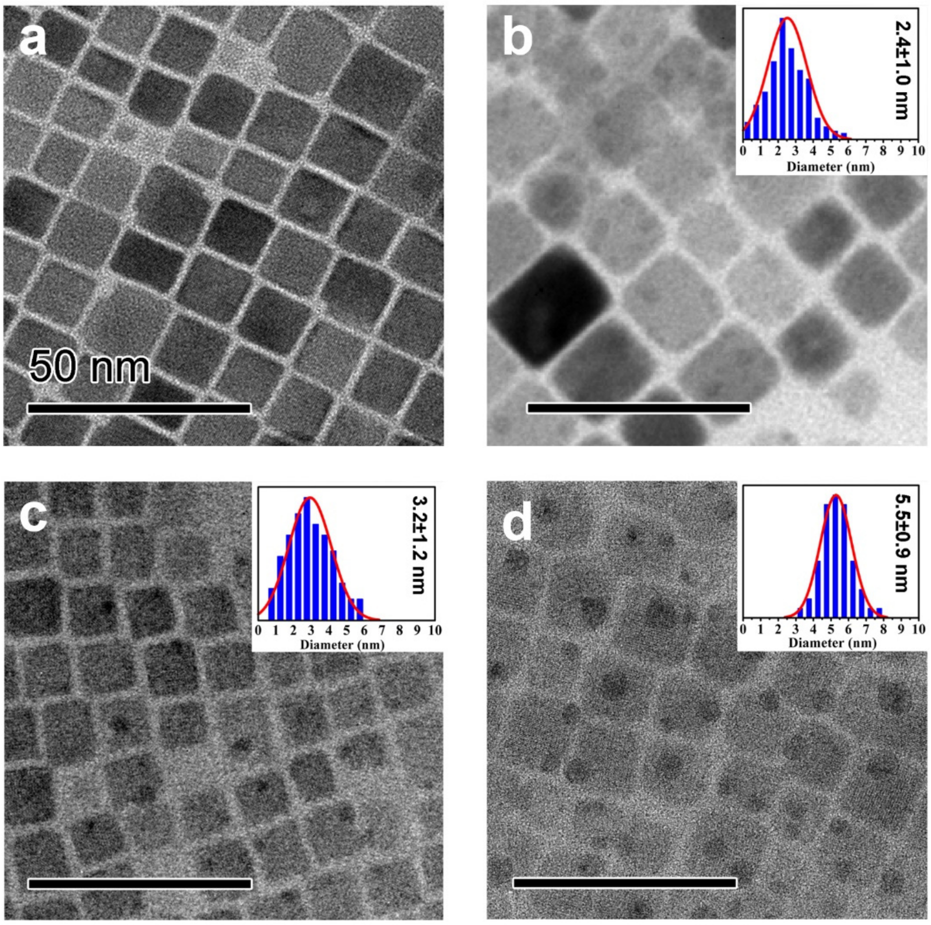


**Supplementary Figure 1 | Ripening process of Ag nanoparticles**. Typical TEM images of Ag–$CsPbBr_3$ hybrid nanocrystals produced with different irradiation time: (**a**) 0, (**b**) 5, (**c**) 30, and (**d**) 60 min. Scale bar: 50 nm. The insets are the size distributions of the corresponding Ag nanoparticles. The sample (**d**) is employed in this study.

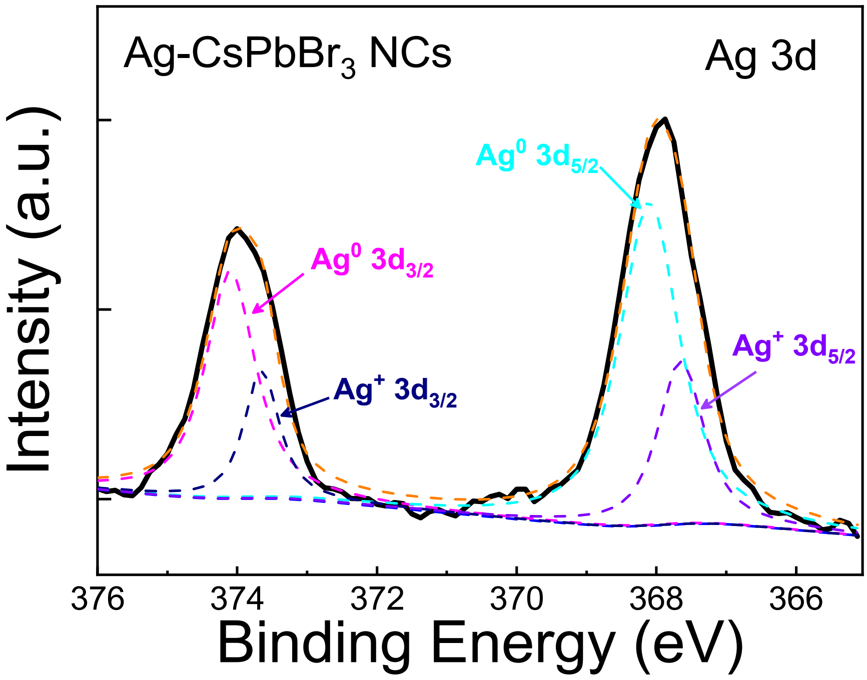


**Supplementary Figure 2 | High-resolution X-ray photoemission spectrum of Ag 3d band of Ag–$CsPbBr_3$ nanocrystals.** The ratio of $Ag^0$ and $Ag^+$ in samples can be extracted from the X-ray photoemission spectrum by determined peak positions of $Ag^0$ and $Ag^+$.[1] $Ag_2O$ or AgBr is possibly induced during the sample preparation and the X-ray photoemission measurements. In addition, optical absorptions of $Ag_2O$ and AgBr have no peaks in the wavelength range we studied.

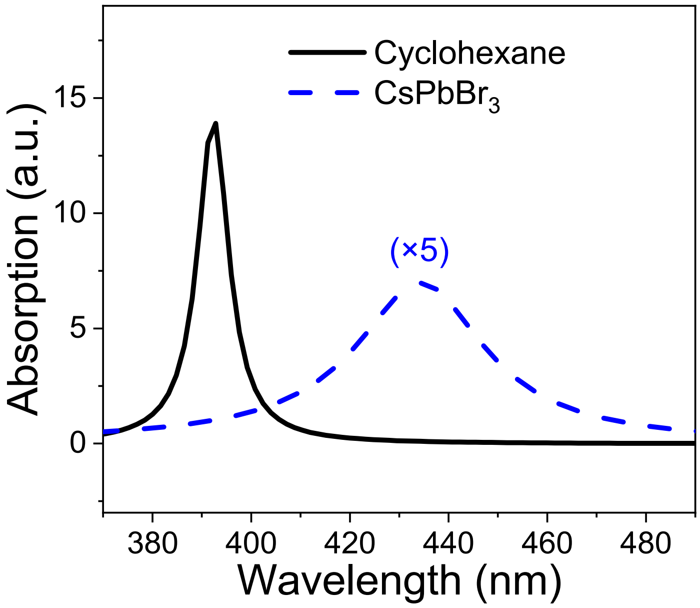


**Supplementary Figure 3 | Simulation of plasmon absorption of Ag nanoparticles.** The calculated absorption spectrum of Ag nanoparticles in cyclohexane shows a peak at 390 nm with a full-width-at-half-maximum (FWHM) of ~ 64 meV, and that of Ag nanoparticles embedded in $CsPbBr_3$ shows a peak at 430 nm with a FWHM of ~ 150 meV. The plasmon absorption measured in Ag–$CsPbBr_3$ NCs in this work is between these two values because the Ag nanoparticles are on the surface of $CsPbBr_3$ NCs. (The theoretical model and the refractive index of silver were from previous literature,[2, 3] and the real and imaginary part of refractive index of $CsPbBr_3$ are estimated to be n=1.8 and k=0.13 through the above spectrum range.[4])

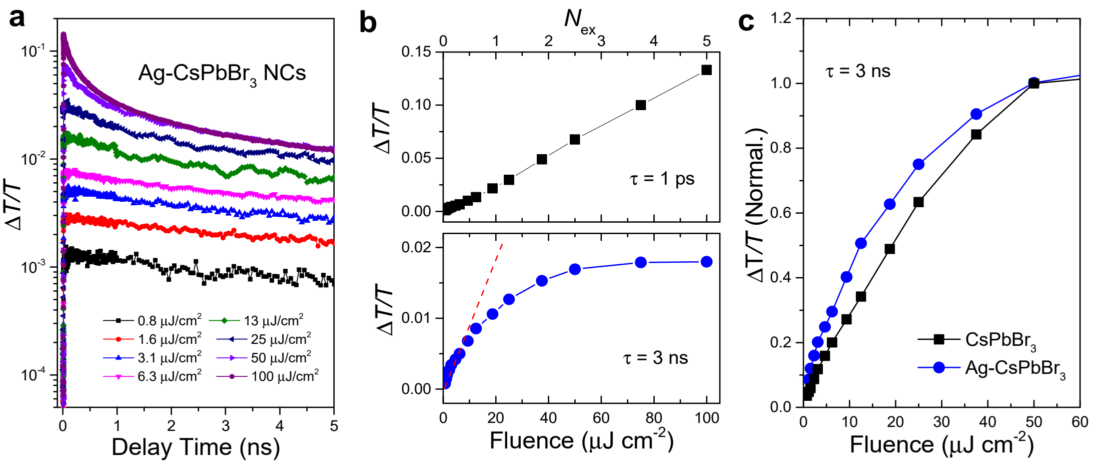


**Supplementary Figure 4| Exciton density dependence of carrier dynamics.** (**a**) Fluence-dependent kinetic dynamics of Ag-$CsPbBr_3$ hybrid nanocrystals probed at 514 nm. (**b**) The signal amplitudes probed at time delays of 1 ps and 3 ns are plotted versus pump fluence (average number of excitons per dot) in Ag-$CsPbBr_3$ hybrid nanocrystals. The dashed line is the curve fitted to a linear function for the data recorded under weak excitations. (**c**) The power dependences of signal amplitudes at the delay of 3 ns for Ag-$CsPbBr_3$ and $CsPbBr_3$ nanocrystals are compared in a scale normalized at the amplitude recorded at 50 μJ $cm^{-2}$.

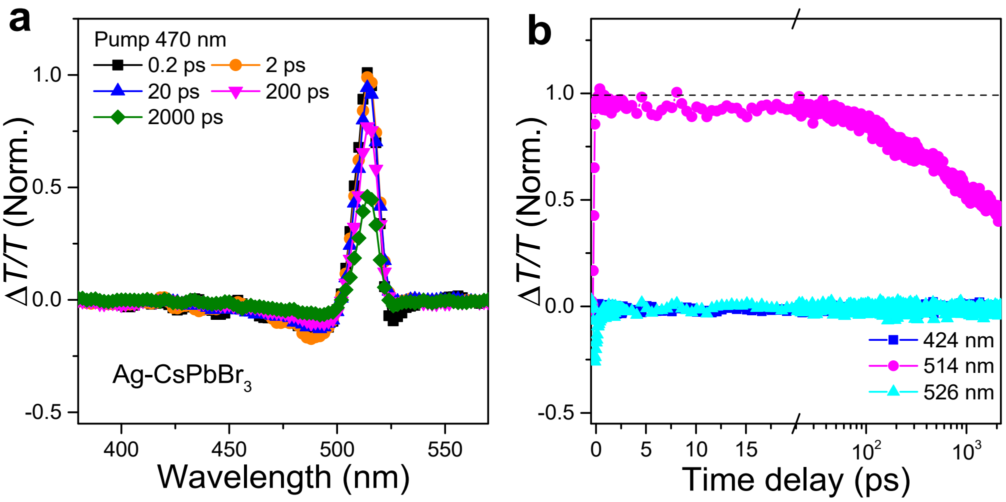


**Supplementary Figure 5 | TA data for Ag–$CsPbBr_3$ nanocrystals under pumping at 470 nm.** (**a**) TA spectra recorded at different time delays. (**b**) Kinetic curves probed at different wavelengths.

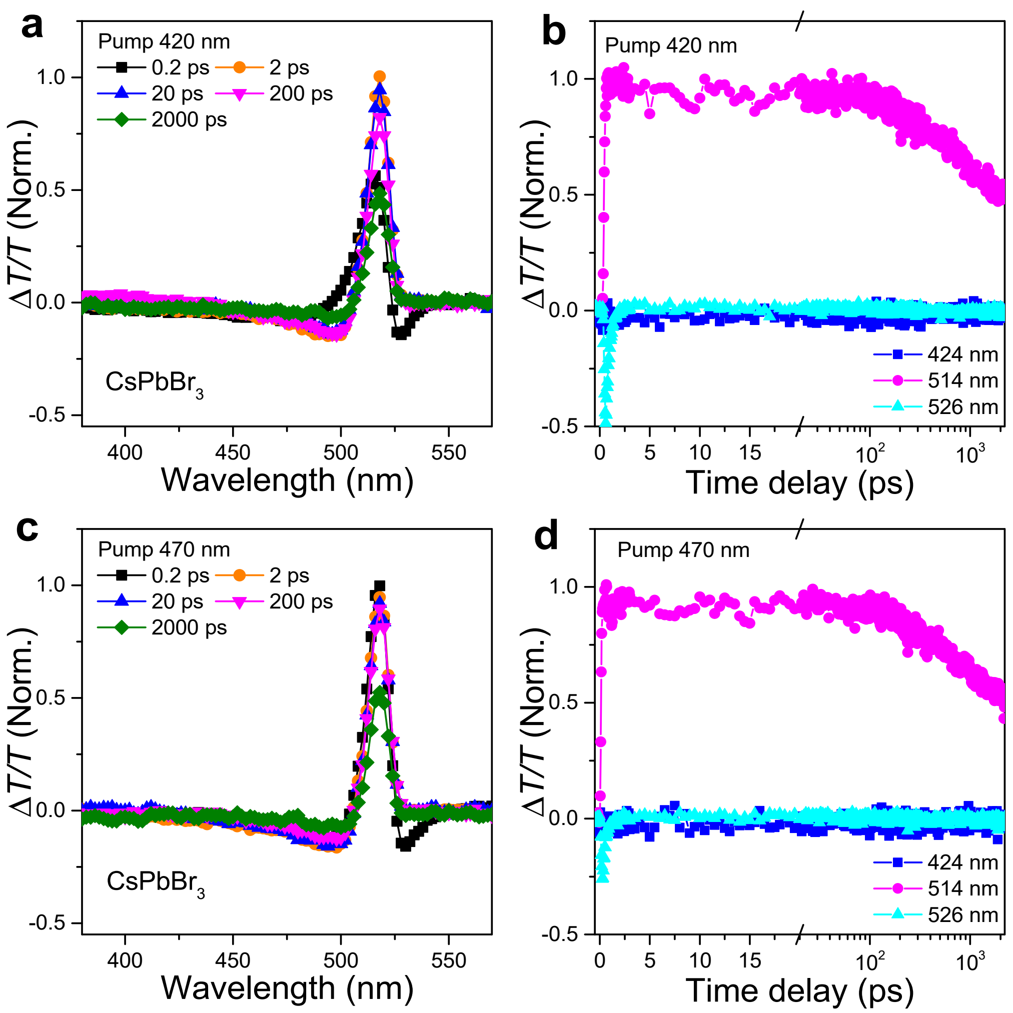


**Supplementary Figure 6 | TA spectra for a solution sample of neat $CsPbBr_3$ nanocrystals**. (**a**) TA spectra recorded from neat sample of $CsPbBr_3$ nanocrystals at different time delays under pump at 420 nm. (**b**) Kinetic curves probed at different wavelengths pumped at 420 nm. (**c**, **d**) are the same as (**a**, **b**) but pumped at 470 nm.

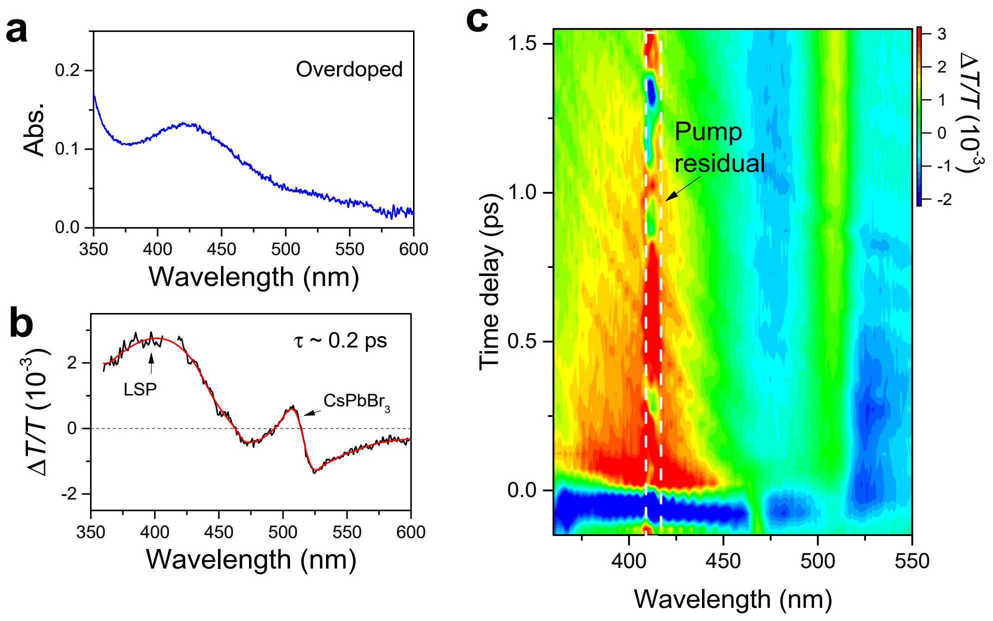


**Supplementary Figure 7 | Carrier dynamics in an overdoped sample.** (**a**) The absorption spectrum and (**b**) TA spectrum at the time delay of 0.2 ps of the overdoped sample. The bleach signal in the range < 450 nm is contributed by local mode of surface plasmon (LSP). (**c**) The TA data of the overdoped sample are plotted versus time delay and probe wavelength.

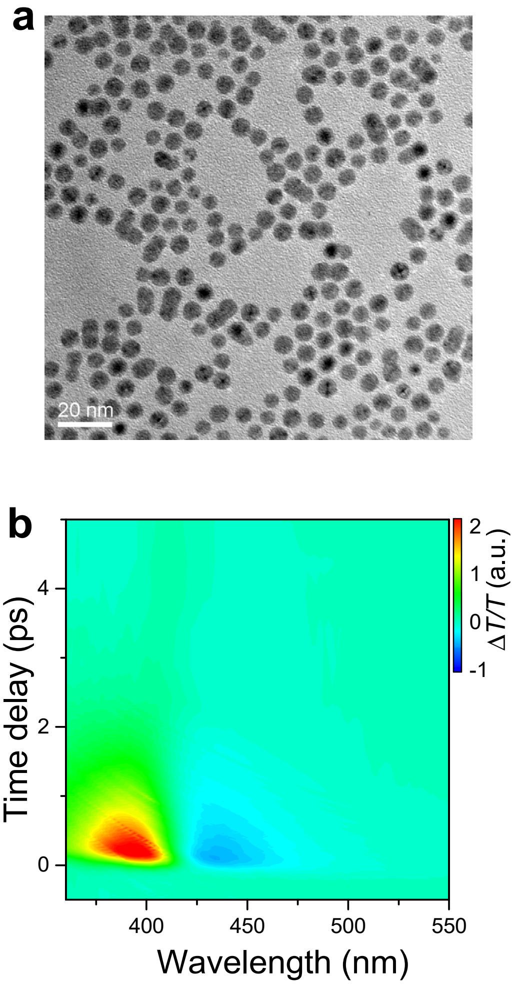


**Supplementary Figure 8 | TA data for neat Ag nanoparticles**. (**a**) TEM image of Ag nanoparticles. (**b**) TA spectral data for Ag nanoparticles. The pump wavelength was 390 nm that is resonant to the local mode of surface plasmon in neat Ag nanoparticles.

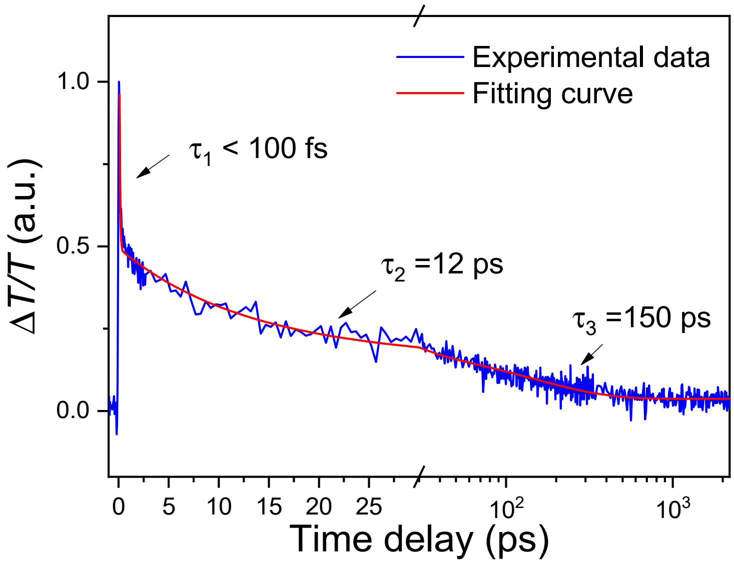


**Supplementary Figure 9 | Complete dynamics of Ag–CsPbBr$_3$ nanocrystals**. The sample was pumped at 420 nm and probed at 424 nm, the wavelength of local mode of surface plasmon. The dynamics is fitted with three decay components.

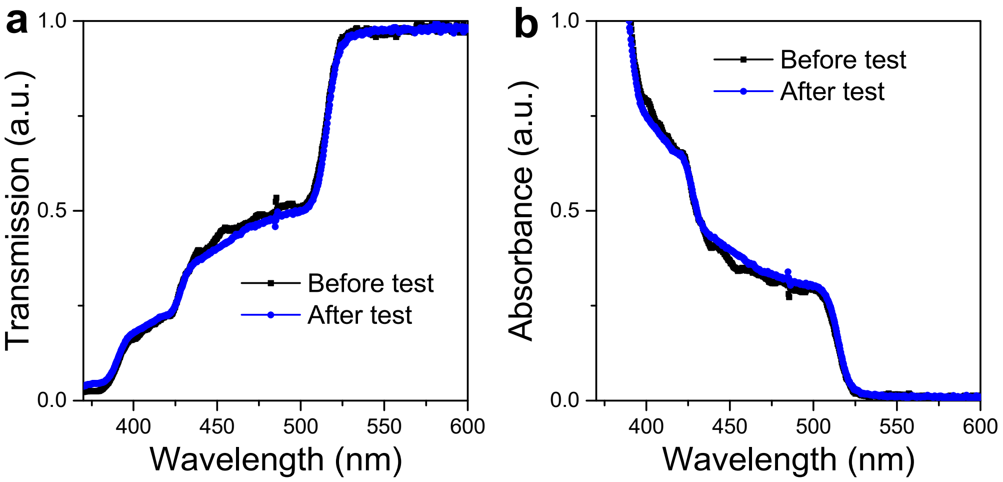


**Supplementary Figure 10 | Sample stability confirmation.** The transmission spectra (**a**) and absorption spectra (**b**) of a solution sample before and after TA measurements. The absorption spectra show considerably agreement. The spectra were measured using supercontinuum as the light source. The slight disparity in the two curves is possibly related to the instability of the supercontinuum.

**Supplementary Note 1. Fluence-dependent carrier dynamics in Ag-$CsPbBr_3$ NCs.**

In semiconductor nanocrystals (NCs), Auger recombination becomes significant under high-fluence pump due to the quantum confinement effect.[5] In $CsPbBr_3$ NCs, such a many-body effect has been reported in literatures.[6-8] In Supplementary Figure 4, we show the carrier dynamics in Ag-$CsPbBr_3$ NCs recorded under different pump fluences. The Auger recombination is observed with saturated signal amplitude at 3 ns and faster recombination under high-fluence pump. To avoid Auger recombination in our analysis, we present the data recorded with low fluence pump (3 μJ $cm^{-2}$). The average number of photons absorbed by a nanocrystal is ~ 0.2. In the regime, the many-body effect is negligible since most of excited nanocrystals absorb only one photon in the approximation of Poisson distribution.

**Supplementary Note 2. Quantification of hot excitation transfer in Ag-$CsPbBr_3$ NCs.**

Both plasmon-induced resonant energy transfer (PIRET) and hot-electron transfer (HET) channels are present in the hybrid sample of Ag-$CsPbBr_3$ NCs. To quantify the quantum efficiencies of the channels, it is necessary to evaluate the population of excited states per absorbed photons. Two types of excited states may be formed in the hybrid systems: the electron-hole pair (EHP) and the interfacial charge-separated state. Under off-resonant pump, only the EHP is created by direct optical absorption. Under resonant pump, the excited states of semiconductor may be formed with the EHP created by direct absorption, the EHP induced by PIRET, or the charge-separated state caused by HET. Photoluminescence emission is mainly induced by the EHP. From photoluminescence excitation spectra, PIRET induces $\eta_1$~ 15±5% increment of EHP when SPR is resonantly excited. TA signal includes the contributions from the EHP and the charge-separated state population. As discussed in the text, the bleach signal of a charge-separated state population is about a half of that of an EHP. The XB signals per absorbed photon probed at 514 nm under resonant (420 nm) and off-resonant (470 nm) pumps can thus be described as,

$$XB(420\,\mathrm{nm})/N_{\mathrm{PH}}(420\,\mathrm{nm}) \propto N_{\mathrm{EHP}} + \eta_1 N_{\mathrm{EHP}} + N_{\mathrm{CS}}/2, \quad (1)$$

$$XB(470\,\mathrm{nm})/N_{\mathrm{PH}}(470\,\mathrm{nm}) \propto N_{\mathrm{EHP}}. \quad (2)$$

Here, $XB(\lambda), N_{\mathrm{PH}}(\lambda)$ are the XB signal amplitudes and the photon density absorbed by the sample at the incident wavelength $\lambda$; $N_{\mathrm{EHP}}$ and $N_{\mathrm{CS}}$ are the populations of EHPs created by optical absorption and charge-separated states induced by HET, respectively. With the above equations, we can calculate the charge-separated state population induced by plasmonic absorption to estimate the quantum efficiency of HET.

To minimize the effect of errors when measuring the pump intensity, we use the signals from a control sample of neat $CsPbBr_3$ NCs as reference. The ratio of enhanced absorption ($\Delta_{\mathrm{abs}}(\lambda)$) can be quantified as,

$$\Delta_{\mathrm{abs}}(\lambda) = \frac{(1-10^{-OD_{\mathrm{Ag\text{-}CPB}}(\lambda)}) - (1-10^{-OD_{\mathrm{CPB}}(\lambda)})}{(1-10^{-OD_{\mathrm{CPB}}(\lambda)})}, \quad (3)$$

where $OD_{\text{Ag-CPB}}(\lambda)$ and $OD_{\text{CPB}}(\lambda)$ are the absorbance of the Ag–$CsPbBr_3$ NCs and neat $CsPbBr_3$ NCs at the wavelength λ, respectively. As discussed above, the plasmon-induced XB signal can be obtained by subtracting the XB signal containing the contribution from $CsPbBr_3$ ($XB_{\text{CPB}}$). The enhancement ratio of TA signal ($\Delta_{\text{sig}}(\lambda)$) can be thus estimated as

$$\Delta_{\text{sig}}(\lambda) = \frac{XB_{\text{Ag-CPB}}(\lambda) - XB_{\text{CPB}}(\lambda)}{XB_{\text{CPB}}(\lambda)}, \tag{4}$$

where $XB_{\text{Ag-CPB}}(\lambda)$ and $XB_{\text{CPB}}(\lambda)$ are the XB signal amplitudes of Ag–$CsPbBr_3$ NCs and neat $CsPbBr_3$ NCs at a pump wavelength of $\lambda$, respectively. The above approach is equivalent to the method used in the literature.[9] However, using the data from control sample of $CsPbBr_3$ NCs can eliminate the errors caused by measuring the sample concentrations and pump intensity.

From the trace normalized to the signal amplitude at the delay of 2 ns, $\Delta_{\text{sig}} / \Delta_{\text{abs}}$ is ~ 35±15% at the delay of 50 ps with pump at 420 nm. Considering the EHPs induced by PIRET, the normalized signal increment can be expressed as,

$$\Delta_{\text{sig}} = \frac{\eta_1 N_{\text{EHP}} + N_{\text{CS}} / 2}{(1+\eta_1) N_{\text{EHP}}}. \tag{5}$$

The quantum efficiency of HET can be calculated as the population of CS state per photon absorbed by plasmon, i.e.,

$$\eta_2 = \frac{N_{\text{CS}} / N_{\text{EHP}}}{\Delta_{\text{abs}}}. \tag{6}$$

The value is estimated to be ~ 50 ± 18%. The errors in quantifying the quantum efficiencies are standard deviations obtained by averaging several measurements.

## Supplementary References


1. Zhu, X., Lee, J. & Lu Wei, D. Iodine vacancy redistribution in organic–inorganic halide perovskite films and resistive switching effects. *Adv. Mater.* **29**, 1700527 (2017).
2. Papavassiliou, G. C. Optical properties of small inorganic and organic metal particles. *Prog. Solid State Chem.* **12**, 185-271 (1979).
3. Wu, Y. *et al.* Intrinsic optical properties and enhanced plasmonic response of epitaxial silver. *Adv. Mater.* **26**, 6106-6110 (2014).
4. Yakunin, S. *et al.* Low-threshold amplified spontaneous emission and lasing from colloidal nanocrystals of caesium lead halide perovskites. *Nature Commun.* **6**, 8056 (2015).
5. Klimov, V. I. *et al.* Quantization of multiparticle Auger rates in semiconductor quantum dots. *Science* **287**, 1011-1013 (2000).
6. Yarita, N. *et al.* Dynamics of charged excitons and biexcitons in $CsPbBr_3$ perovskite nanocrystals revealed by femtosecond transient-absorption and single-dot luminescence spectroscopy. *J. Phys. Chem. Lett.* **8**, 1413-1418 (2017).
7. Xu, Y. *et al.* Two-photon-pumped perovskite semiconductor nanocrystal lasers. *J. Am. Chem. Soc.* **138**, 3761-3768 (2016).
8. Makarov, N. S. *et al.* Spectral and dynamical properties of single excitons, biexcitons, and trions in cesium–lead-halide perovskite quantum dots. *Nano Lett.* **16**, 2349-2362 (2016).
9. Wu, K., Rodriguez-Cordoba, W. E., Yang, Y. & Lian, T. Plasmon-induced hot electron transfer from the Au tip to CdS rod in CdS-Au nanoheterostructures. *Nano Lett.* **13**, 5255-5263 (2013).
